# ENCORE! Getting to the core of the ischemic core at the core lab

**DOI:** 10.3389/fstro.2024.1389830

**Published:** 2024-04-05

**Authors:** David S. Liebeskind, Scott Brown, Albert J. Yoo

**Affiliations:** ^1^Neurovascular Imaging Research Core, University of California, Los Angeles, Los Angeles, CA, United States; ^2^Comprehensive Stroke Center and Department of Neurology, David Geffen School of Medicine at the University of California, Los Angeles, Los Angeles, CA, United States; ^3^BRIGHT Research Partners, Mooresville, NC, United States; ^4^Division of Neurointervention, Texas Stroke Institute, Dallas-Fort Worth, TX, United States

**Keywords:** stroke, imaging, endovascular therapy, ischemic core, collaterals

## Abstract

The recent results of large core trials in acute ischemic stroke prompt the most influential paradigm shift in over a decade since endovascular therapy (EVT) was proven as the mainstay of acute stroke treatment. Six randomized, controlled trials (RCTs) of EVT in patients with more extensive ischemia on imaging than prior approved treatment indications revealed a consistent benefit of EVT, despite different imaging criteria and ischemic core definitions across the individual trials. These findings unfolded sequentially, akin to the acts of a drama, with the story evolving until perhaps the most conclusive results on extensive ischemia treatment about to be published. At this juncture, it is important to summarize the collective findings of these 6 RCTs, consider the potential expansion in the use of EVT, outline the penultimate or next steps during the encore of this drama and ponder the broad implications of these recent findings on the entire stroke field for many years to come. This logical rationale and perspective form the basis of Establishing Neuroimaging Criteria of Revascularization Efficacy (ENCORE), the proposed pooled, subject-level, meta-analysis of the recent large core trials, leveraging imaging re-adjudication by a collaborative, imaging core lab with documented expertise. Expansion of the treatment indications for EVT will likely ensue, yet numerous questions persist regarding other confounding variables that we normally use in routine clinical practice. Precision medicine for large core ischemic stroke mandates a more detailed investigation of the numerous clinical, imaging and angiographic variables that each trial dataset collected. In a simple pooled meta-analysis, these varying definitions, methodology and analytic approaches preclude anything beyond current summary results showing that EVT is favored, in general, over medical treatment. The epilog of the large core trials can be written by ENCORE, using the imaging and statistical methods and relevant expertise of the prior Highly Effective Reperfusion Evaluated in Multiple Endovascular Stroke Trials (HERMES) collaboration.

## Introduction

The six recent large core trials include: Recovery by Endovascular Salvage for Cerebral Ultra-Acute Embolism–Japan Large Ischemic Core Trial (RESCUE-Japan LIMIT) (Yoshimura et al., 2022); Endovascular Therapy in Acute Anterior Circulation Large Vessel Occlusive Patients with a Large Infarct Core (ANGEL-ASPECT) (Huo et al., 2023); Randomized Controlled Trial to Optimize Patient's Selection for Endovascular Treatment in Acute Ischemic Stroke (SELECT 2) (Sarraj et al., 2023); Thrombectomy for Emergent Salvage of Large Anterior Circulation Ischemic Stroke (TESLA) (ESOC., 2023); The Efficacy and Safety of Thrombectomy in Stroke with extended lesion and extended time window (TENSION) (Bendszus et al., 2023); Large Stroke Therapy Evaluation (LASTE) (Costalat et al., 2024). The timing of the presentation and publication of each trial key results were intermixed, and the story arc of large ischemic core stroke patient outcomes grew complex over the last 2 years. Each trial employed somewhat different design elements and methodology across varying geographical regions, with overall results favoring EVT over medical treatment alone, yet distinctive features of each trial may prompt slightly different interpretation and ultimate impact on the future treatment of stroke. RESCUE-Japan LIMIT enrolled 203 patients with Alberta Stroke Program Early Computed Tomographic Score (ASPECTS) scores of 3 to 5 on baseline imaging, demonstrating better functional outcomes with EVT despite more frequently associated hemorrhagic transformation (HT), although MRI was used for diagnosis in 175/203 (86%) (Yoshimura et al., 2022). ANGEL-ASPECT used combinations of non-contrast CT ASPECTS and/or CT perfusion (CTP) infarct core volumes that varied by time window in 456 patients, revealing no additional impact of CTP core delineation on patient selection within 24 h of symptom onset (Huo et al., 2023). SELECT 2 enrolled 352 patients with CT ASPECTS of 3–5 or infarct core volumes ≥50 ml on CTP in 343/352 (97%), with vascular complications noted in 18.5% of EVT cases and remarkably low rates of symptomatic HT (Sarraj et al., 2023). A recent correction clarified a variety of imaging methods used, including conflicting definitions of ischemic core volumes from those prespecified for determination of large core at enrollment (Sarraj et al., 2024). TESLA adhered to low ASPECTS (2–5) on noncontrast CT within 24 h in 300 patients, showing better functional outcomes after EVT, although the pre-specified efficacy endpoint was not reached (ESOC., 2023). TENSION predominantly (82%) used non-contrast CT with ASPECTS 3–5 in 253 patients within 12 h of symptom onset and demonstrated efficacy of EVT with lower mortality than medical treatment alone (Bendszus et al., 2023). LASTE enrolled 324 patients with ASPECTS 0–5 (median 2) with MRI in 84% within 6.5 h of symptom onset to reveal better outcomes and lower mortality after EVT (Costalat et al., 2024).

Unlike other pooling of individual RCT results, the primary outcomes were consistent across all individual studies, demonstrating the clear benefit of EVT over medical treatment alone in large core strokes. However, the heterogeneous use and combinations of basic to advanced imaging modalities for trial selection criteria and imaging methods regarding treatment outcomes, including hemorrhagic transformation (HT), complicate the broader implementation of EVT around the world for ischemic strokes of any size, compounded by discrepant time windows for potential intervention used in each of the RCTs. Expansion of the treatment indications for EVT will likely ensue, yet numerous questions persist regarding other confounding variables that we normally use in routine clinical practice. Precision medicine for large core ischemic stroke mandates a more detailed investigation of the numerous clinical, imaging and angiographic variables that each trial dataset collected. In a simple pooled meta-analysis, these varying definitions, methodology and analytic approaches preclude anything beyond current summary results showing that EVT is favored, in general, over medical treatment. The associated complications and impact on workflow, resource utilization or costs also remain unknown.

The epilog of the large core trials can be written by ENCORE, using the imaging and statistical methods and relevant expertise of the prior HERMES (Highly Effective Reperfusion Evaluated in Multiple Endovascular Stroke Trials) collaboration (Goyal et al., 2016). In HERMES, the original imaging source data across all modalities (e.g., multimodal CT or MRI, conventional angiography during EVT) from each RCT was compiled, blinded to trial source, treatment allocation and any clinical or outcome data. Imaging was then re-adjudicated by expert core lab readers using standardized definitions and methods. The central statistical expert integrated these resultant imaging data with the pooled, clinical trial datasets to run a series of analyses that examined particular facets of the greater dataset. ENCORE may leverage this specific expertise and groundbreaking methodology used in the prior landmark EVT trials to answer many more questions on a topic where imaging definitions are critical. The clarification of large ischemic core definitions is warranted. The detailed angiographic variables such as collateral status, degree of reperfusion and complications are lacking across trials. Specific arterial occlusion site, EVT techniques and even device use also remains unknown, at present. HT subtypes, combinations of such HT subtypes, and the relationship with neurological symptoms equivalently determined across all trials require such analyses. Simple pooling of the 6 RCT final datasets cannot reliably accomplish any of these goals.

The insights derived from ENCORE will have broad implications. For clinical practice, it will provide important estimates of treatment effect based on standardized imaging characteristics in combination with clinical factors such as time window and age. It will yield a better understanding of the pathophysiology of large core stroke by analyzing the imaging evolution of infarcts under prolonged ischemia (medical treatment alone) and after endovascular reperfusion. This may help to identify targets for edema-reducing therapies and neuroprotection. The role of automated imaging pipelines, diverse software packages and even novel analyses, such as mapping of net water uptake in these edematous brains, may be investigated and correlated with age or sex differences, duration of ischemia, concomitant use of thrombolytics, hemicraniectomy and the subtypes of HT in this distinct population of stroke patients previously unstudied with EVT, controlling for medical treatment alone. Such pooling and re-adjudication of 1,890 cases with serial imaging and angiography during EVT in almost a thousand cases is feasible via the imaging core lab used in HERMES. Limitations undoubtedly exist with use of some current imaging core lab services. In clinical practice, the timeliness of imaging interpretation at a core lab, the process of interpretation verification and the management of data transmission need attention. Standardization and optimization of imaging and angiography core lab services is critical for use in future clinical trials, registries and post-marketing surveillance.

The precision medicine of how to optimally treat specific stroke cases with EVT and the future use of adjunctive drugs will be outlined by the course of these relatively large subsets of cases. Such large strokes undergoing thrombectomy are the ideal population to study and ultimately prove the role of neuro- or cytoprotection. Until then, much can be learned about how to organize stroke care (e.g., personnel, timing, imaging resources, expertise) for this rapidly evolving entity of large core strokes around the globe.

## Data availability statement

The original contributions presented in the study are included in the article/supplementary material, further inquiries can be directed to the corresponding author.

## Author contributions

DL: Conceptualization, Methodology, Project administration, Resources, Supervision, Writing – original draft, Writing – review & editing. SB: Data curation, Investigation, Methodology, Writing – review & editing. AY: Conceptualization, Investigation, Writing – review & editing.
